# Supplementary material for: The Perceptions and Use of Urban Neighborhood Parks Since the Outbreak of COVID-19: A Case Study in South Korea
Source: Int J Environ Res Public Health. 2023 Feb 27;20(5):4259. doi: 10.3390/ijerph20054259 (PMC10002250; doi:10.3390/ijerph20054259)
Supplement: Supplementary file 1 [file ijerph-20-04259-s001.zip › ijerph-2197971-supplementary.pdf]

Survey on frequency of visits to neighborhood parks since the outbreak of COVID-19

Good afternoon

This survey is conducted as part of the Korea University Graduate School of Landscape Analysis and User Behavior lecture.

In the past two years, as many changes have occurred in daily life and the threat of infection has increased due to COVID-19, the demand for visits to parks has increased as parks are perceived as relatively low in risk of infection and have fewer restrictions on physical activity and social interaction. Therefore, this survey is conducted to find out the relationship between COVID-19 stress and the motivation and frequency of visiting neighborhood parks. Please answer each question honestly.

Your responses will be kept confidential and will not be used for any purpose other than research data. Also, there is no right or wrong answer to this survey, so please feel free to answer based on your experiences and feelings.

Thank you for taking time out to fill out the survey.

◑ Survey target: People who have experience using neighborhood parks

◑ Survey contents: COVID-19 stress, motivation for visiting neighborhood parks, and frequency of visits

◑ Estimated time for survey: About 5 minutes

* Neighborhood parks: Neighborhood parks are a type of city parks and include the following types of parks. (Article 4 of the Enforcement Rules of the Urban Parks Act)

- Living area neighborhood park: Parks that are mainly intended to be used by people residing in the neighborhood

- Neighborhood Parks within Walking Distance: Neighborhood parks for the purpose of serving people who mainly live within walking distance

- Neighborhood parks in urban easement areas: Neighborhood parks that aim to serve the comprehensive use of all residents residing in urban areas

- Metropolitan Neighborhood Parks: Neighborhood parks that aim to serve a wide range of uses that exceed one urban area

2021. 12.

Section: COVID-19 Stress

Please check the level of stress respondents felt due to COVID-19 in their daily life.

1-1. I am worried about when and where I will be infected with COVID-19.

1-2. I am worried about getting infected with COVID-19 in an enclosed place that I use frequently (e.g., elevators, public transportation).

1-3. I am worried that others will be infected with COVID-19 because of me.

1-4. I'm worried about getting infected with COVID-19 because of people around me.

1-5. I am worried that I will get severely ill from COVID-19.

1-6. I am afraid of losing my livelihood due to COVID-19.

1-7. I'm worried that I might get infected with COVID-19 if I touch a handle in a public place.

1-8. I am worried about getting infected with COVID-19.

1-9. When COVID-19 news is updated on TV or social media, it is stressful.

1-10. Daily life has become terrifying due to COVID-19.

1-11. It is hard to see family and friends very often because of COVID-19.

1-12. Due to COVID-19, more time at home has lowered my will to live and made me lethargic.

1-13. As social distancing continues for a long time, I feel disconnected from society.

1-14. I am depressed because I cannot do hobbies or cultural activities as I did before because of COVID-19.

1-15. I follow the quarantine rules well, but I get angry when other people do not follow them properly.

1-16. I get angry at my boss, seniors, and adults in my family for forcing me to a dinner or meeting without considering the possibility of COVID-19 transmission.

1-17. I get angry when I see people going to high-risk facilities (e.g., pubs, clubs) where there is a risk of spreading COVID-19.

1-18. I get angry at religious people who insist on engaging in contact activities.

1-19. It is difficult to get enough sleep due to the psychological pressure of COVID-19.

1-20. I have been contemplating suicide because of COVID-19 stress.

1-21. It is hard to concentrate because I'm worried about COVID-19.

1-22. When I think of COVID-19, I sweat or my heart beats quickly.

Section: Motivation to park visits

Please check the extent to which the content of the question has influenced your motives for visiting neighborhood parks.

2-1. Because you can go for a walk or jog

2-2. To walk pets

2-3. To exercise in the park

2-4. Because you can breathe the fresh air

2-5. To see vegetation such as trees and flowers

2-6. To experience nature

2-7. To enjoy the beautiful scenery

2-8. To rest

2-9. To energize

2-10. To relieve stress in daily life

2-11. For psychological stability and relaxation

2-12. To read a book in the park

2-13. To take pictures in the park

2-14. For learning

2-15. To meet friends

2-16. To enhance family ties

2-17. To meet new people

2-18. A visit to the park relieves tension

2-19. To satisfy the desire to go out

2-20. Because the park's population density is low.

2-21. To prevent disease through healthy living

2-22. To get out of the house

2-23. Because the natural open space feels safe

2-24. Because the park is sanitary

2-25. Because the park is safer from COVID-19 than other places

2-26. No limit on the number of people

2-27. More free time because daily life has shifted to working at home or online

Section: Change in frequency of neighborhood park visits

Please check the extent to which the frequency of visits to neighborhood parks has changed since the outbreak of COVID-19.

3-1. Frequency of visits to parks before the outbreak of COVID-19

- ○ Never visited
- ○ 1~2 times a week
- ○ 3~4 times a week
- ○ 5days or more a week

3-2. Frequency of visits to parks after the outbreak of COVID-19

- ○ Never visited
- ○ 1~2 times a week
- ○ 3~4 times a week
- ○ 5days or more a week

3-3. If there has been a change in visit frequency, why? (Optional)

Section: Demographic information

4-1. Sex

- ○ Male
- ○ Female

4-2. Age

- ○ 10s
- ○ 20s
- ○ 30s
- ○ 40s
- ○ 50s
- ○ Over 60

4-3. Distance from the neighborhood park you usually visit

- ○ Within 5 mins on foot
- ○ Within 10 mins on foot
- ○ Within 30 mins on foot

- ◯ Over 30 mins on foot

4-4. Work patterns

- ◯ Commuting
- ◯ Work from home
- ◯ Parallel
